# Towards a sustainable model for a digital learning network in support of the Immunization Agenda 2030 –a mixed methods study with a transdisciplinary component

**DOI:** 10.1371/journal.pgph.0003855

**Published:** 2024-12-31

**Authors:** Isis Umbelino-Walker, Ana Paula Szylovec, Brice Alain Dakam, Asta Monglo, Ian Jones, Charlotte Mbuh, Reda Sadki, Alan Brooks

**Affiliations:** 1 Bridges to Development, Geneva, Switzerland; 2 Movement for Immunization Agenda 2030 (IA2030), Buea, Cameroon; 3 South West Regional Fund for Health Promotion, Buea, Cameroon; 4 The Geneva Learning Foundation, Geneva, Switzerland; 5 Movement for Immunization Agenda 2030 (IA2030), Maroua, Cameroon; 6 Independent, Maroua, Cameroon; King's College London, UNITED KINGDOM OF GREAT BRITAIN AND NORTHERN IRELAND

## Abstract

The Immunization Agenda 2030 (IA2030) has been endorsed at the World Health Assembly as the world’s strategy for immunization. The Movement for IA2030 is a voluntary collective of immunization practitioners, principally from low- and middle-income countries, who have pledged to support each other to accelerate local action in support of this global strategy. Collective action is facilitated by the peer learning platform established by The Geneva Learning Foundation (TGLF). We reviewed existing data from Movement participants and collected additional survey data to explore two aspects of sustainability of the Movement: why immunization practitioners participate and how much time and resources they contribute. Quantitative analyses of an existing large data set (n = 5682 participants) were complemented by analyses of data collected through a new survey (n = 291) and focus groups of Movement participants. The most commonly cited reason for participating (32% of responses) was to share experience and learn from others. This was generally true across all levels of experience, gender and place of work, suggesting a common rationale among immunization practitioners in different settings. It was a particularly strong motivation for the most experienced practitioners and those working in a setting with a strong organizational learning culture. New survey data revealed a high degree of intrinsic personal motivation to participate, through commitment of significant time and financial resources, with 47% (n = 136) of respondents committing personal finances to implement an action plan. Focus group discussions provided insights into the implications of findings for sustaining the Movement. Collectively, the analyses highlight key aspects of voluntary collective action to achieve global immunization goals through local action, to inform efforts to ensure long-term sustainability of the Movement.

## Introduction

The Immunization Agenda 2030 (IA2030), the global immunization strategy for 2021–2030, has set ambitious targets for global immunization coverage and other key indicators [1]. In part because of disruptions caused by the COVID-19 pandemic, the world is currently off-track to achieve these targets [2]. IA2030 highlights several challenges for immunization in countries and communities worldwide, including: human resource gaps; limitations in the quality and use of immunization and surveillance data; coverage monitoring; uneven access to vaccines at affordable prices; emerging inequities in middle-income countries; inadequate and unpredictable financing; outbreaks; conflicts; and humanitarian emergencies [3]. Local health practitioners are frequently on the frontline of managing or mitigating the implications of such challenges. Hence, there is considerable interest in additional approaches that improve vaccination coverage, particularly in low- and middle-income countries (LMICs), where the vast majority of unimmunized and under-immunized children live [4].

At the 74th World Health Assembly in May 2021, the Director-General of the WHO, Dr Tedros Adhanom Ghebreyesus, called for “a broad social movement for immunization that will ensure that immunization remains high on global and regional health agendas and help to generate a groundswell of support or social movement for immunization” [5].

In response, The Geneva Learning Foundation (TGLF), a Geneva-based non-profit specializing in peer learning programs for healthcare workers in LMICs, developed a learning program intended to contribute to a “Movement for IA2030”. Those joining the Movement made an “Immunization Scholar Pledge for Impact”, signing up to work towards IA2030’s and their country’s goals, to adhere to the IA2030 core principles, and to provide support to their peers making similar commitments [6].

Peer learning enables health practitioners to learn from each other, sharing experience and expertise relating to specific local challenges. This exchange is facilitated (or “scaffolded” as it is known in the education field [7]) by a digital and pedagogical infrastructure developed by TGLF, which provides virtual spaces for peer–peer interactions and evidence-based learning exercises. The peer learning approach for IA2030 aimed to leverage the experience [8] and tacit knowledge [9] accumulated by immunization practitioners.

Digital learning or e-learning platforms have transformed learning opportunities for global health practitioners by offering flexible, accessible, low-cost, and personalized learning experiences. Digital platforms have been tailored for knowledge transfer to tens of thousands of participants [10]. Other platforms have focused on specific topics, sometimes framed as digital communities of practice [11, 12]. Some platforms have specifically included support for peer learning [13, 14]. Platforms are challenged to balance considerations around functioning (e.g. scale, diversity of topics, infrastructure to support peer learning, translation of knowledge into practice) and sustainability.

The TGLF approach draws on recent advances in learning science that leverage new opportunities offered by digital technologies [15], and recognize the complexity of learning networks [16, 17] and the importance of “learning culture”, the extent to which organizations value and encourage continuous learning [18]. Key features of the TGLF peer learning program include problem-based learning (participants apply learning while tackling a key local challenge), peer feedback (sharing experience and offering advice), and digital networking (one-to-one and group dialogue) that meets participants on the platforms of their choice (for example, synchronously in Zoom at live experience-sharing events and asynchronously through recordings, multiple social media platforms, and private communication on WhatsApp, Telegram, and other instant messaging platforms).

The TGLF peer learning program is structured around a “Full Learning Cycle” (FLC), during which participants undertake a sequential series of learning activities. For 2022, the FLC lasted 14 weeks. Activities began with a diagnostic questionnaire that includes the identification of a key local challenge. This was followed by sharing of ideas and practices in TGLF’s “IA2030 Ideas Engine”, a crowdsourced repository of experiences, advice and tools shared by participants and categorized by IA2030 strategic priority. Each participant then developed a situational analysis to better understand the root causes of their key local challenge. This situational analysis then informed the development of a succinct, locally tailored action plan, which identified corrective actions to be undertaken or facilitated by the individual to address the root causes of their challenge. A project kick-off phase, the “Impact Accelerator” (IA), supported participants during their initial implementation stages, to create momentum for sustained action.

Participants were able to opt into each activity. Additional activities, both public (such as live experience-sharing sessions or asynchronous discussions) and private (such as one-to-one networking and “remote coffees”), helped participants connect to share lessons learned, successes and challenges, and strengthened mutual support and emotional connections between participants. Many participants choose to connect outside of these activities independently. The impact of this approach in the immunization context has been extensively documented by participants and TGLF researchers [19–22], and reported in the peer-reviewed literature [23, 24].

The aim of this study was to explore two specific aspects related to the long-term sustainability of the Movement for IA2030: participants’ rationale for joining the Movement and the personal resource commitments (time and money) made by participants. The study was grounded in the collective action theory [25, 26] concepts as a framework to understand and unravel pathways to sustainability.

## Materials and methods

### Research design

This research was a sequential exploratory mixed-methods study [27] with a transdisciplinary component [28] where quantitative data (from participant surveys) informed the qualitative and transdisciplinary phases (focus groups). The research was divided into three phases: (i) exploratory, (ii) survey, and (iii) transdisciplinary.

For this study, we defined collective action as a group of individuals who are cooperatively and voluntarily addressing a common problem, pursuing a shared goal [25]. In this case, individuals had committed to the “Immunization Scholar Pledge for Impact” [6].

Evaluating collective action for the IA2030 Movement is challenging because of the participation of various actors and their complex interconnections [25, 26, 29]. We adopted concepts of institutional analysis and development (IAD) rooted in Ostrom’s work [25] and adapted by Rahman et al. [29]. IAD is a research framework that spans multiple disciplines [26, 29]. It has been used mainly to assess the capacity of individuals to establish groups and the extent to which these groups possess agency in utilizing resources.

In this study, we drew on three concepts from IAD: social capital, human capital, and financial capital. Social capital is a complex social construct encompassing trust-based relationships, reciprocal interactions, and interconnected social networks. Human capital reflects the knowledge and capacity of community members to engage in effective cross-level communication to facilitate knowledge co-production and translation with other groups across different levels. Financial capital indicates the level of individual resources, including time and financial costs, invested in the development of voluntary collective action [29].

### Phase I: Exploratory

The preparation phase was exploratory and quantitative [27, 30]. The aim was to explore existing data on IA2030 Movement participants’ (i) reasons for joining the Movement and (ii) levels of participant investment. This work also informed the structure of the new survey specific to this study and the instruments for the focus group discussions.

#### Existing data

Two surveys were distributed by TGLF to members of the IA2030 Movement in 2022 at different stages of the Full Learning Cycle. Each asked for explicit consent from the respondent allowing the data to be used for research, learning, evaluation, communication and advocacy. For this phase, we accessed data on 20 January 2023 from the online “Application Survey” (n = 5682 respondents), which consisted of 95 questions, and the “Impact Accelerator Survey” (n = 412 respondents), which consisted of 18 questions [31]. The Application Survey was distributed online via social media so it is unknown how many received it. A total of 1024 people received the Impact Accelerator Survey, disseminated in both English and French. The Application Survey was opened on 11 February 2022, and analyses included data up to the programme launch on 7 March 2022. It enabled participants to join the Movement and participate in the Full Learning Cycle, to commit to the goals the Movement for IA2030, and to reflect on their local situation and challenges. For TGLF, responses guided programme design focused on the contexts and priorities of participants, to help the cohort understand its collective identity (“who we are”) and to answer learning questions of interest to global partners [32]. The Application Survey included an evidence-based measurement of learning culture (the capacity of a work environment to support learning and change) and questions related to motivation, based on two standardized motivation scales [32–34]. The Impact Accelerator Survey was opened from 4–18 July 2022, after the final component of the Full Learning Cycle. It enabled participants who developed action plans to report on short-term progress in implementation.

#### Analysis

Descriptive analyses were conducted on the main reasons given by members for joining the IA2030 Movement and for interacting with other members. This analysis involved examining various statistical measures, including frequency, measures of central tendency (like mean or median), measures of variation (such as standard deviation), and measures of position (like percentiles) to gain insights into the characteristics of the data. Analyses were carried out using Power BI and Excel.

Relationship analyses were carried out to assess the association between participants’ reported reasons for joining the IA2030 Movement and demographic factors (gender, professional experience, health system level) and organizational learning culture at their place of work (e.g. ministry of health, NGO). Organizational learning culture was assessed through questions in the Application Survey based on the abbreviated, validated form of the Dimensions of the Learning Organization Questionnaire (DLOQ) [33]. The variable was then transformed by calculating the average of seven questions, each of which had six options in a Likert-scale. The results were then divided into quartiles to provide a categorization of the learning culture of the participant’s organization (1 to 3.6 Very Low, 3.6 to 4.42 Low, 4.57 to 5 High, 5 to 6, Very High).

Using ML.Net’s L-BFGS algorithm, a logistic regression model was developed to identify associations between the reasons cited by participants for joining the Movement for IA2030 and demographic factors and organizational learning culture [35, 36]. Analyses were carried out using Power-BI visual “Key Influencer”. Statistical significance was set for a p-value less or equal to 0.05.

### Phase II: Sustainability survey

Phase II explored participants’ engagement levels, contributions and investments, nearly a year after the completion of the 2022 Full Learning Cycle.

#### Data collection

We conducted an online survey with a cross-sectional design distributed in English and French. The survey was sent on 11^th^ May 2023 and closed on 18^th^ of May 2023. The content of the survey was informed by phase I–secondary data analysis, iteration with the research team, and insights from TGLF team members. The questionnaire required written informed consent and was divided into four parts: demographics, engagement, contribution and sustainability. The survey combined multiple-choice questions and Likert scales. At the end of the survey, participants were asked if they would like to join a focus group discussion.

The survey was disseminated via e-mail by TGLF to 1191 IA2030 Movement members (570 francophones, 621 anglophones) in LMICs who had committed to achieving the goals of IA2030, developed their local action plan, and been certified by TGLF as having completed at least one component of the 2022 Full Learning Cycle. A total of 349 responses were received (29% response rate). Of these, 51% (n = 176) were from anglophones and 49% (n = 173) from francophones.

#### Analysis

Analysis involved examining statistical measures, including frequency, measures of central tendency (like mean or median), measures of variation (such as standard deviation), and measures of position (like percentiles). Analyses were carried out using PowerBI.

### Phase III: Transdisciplinary

The transdisciplinary component refers to the involvement of IA2030 Movement members to reflect on, interpret and validate preliminary results from phases I and II [27, 28, 30]. This strategy was carried out in two steps. First, a workshop was conducted with the TGLF team to generate insights to inform the design of focus group discussions with Movement members. Second, Movement members attended focus group discussions, one in French and one in English.

#### TGLF workshop

The workshop aimed to seek insights on phase 1 data regarding why Movement members engage and what financial or time contributions they make and. The workshop was held online, via Zoom, on 11 May 2023, for four hours. We recruited via e-mail, following the principles of purposeful sampling, targeting TGLF staff and consultants with significant experience in international governance, voluntary action, global health, and learning science, who are contributing to TGLF development or activities, including the IA2030 Movement. A total of eight individuals provided written consent and participated in the discussion. The workshop was facilitated in English by IU-W and AB. After the workshop discussions, participants contributed to finalizing the preparations for the focus group discussions.

### Focus group discussions

The aim of the online (Zoom) focus group discussions was for IA2030 Movement members to help analyze and interpret the data from phases I and II and to discuss sustainability for the IA2030 Movement.

#### Sample and setting

Participants were recruited via the survey in phase II as described above. For the English survey, 75% (n = 133) of respondents volunteered to take part in a focus group discussion. Of those, 74% (n = 99) were males, and 26% (n = 34) were females. For the French survey, 38% (n = 65) of respondents volunteered to take part in a focus group discussion. Of those, 64% (n = 42) were males, and 23% (n = 23) were females. For both cohorts, data were disaggregated by gender and eight females and seven males were randomly selected for each focus group, and then verified for inclusion of different countries, professions and levels of the health system. Written informed consent was obtained and participants were invited to the online (Zoom) focus groups via e-mail. A number of confirmed participants had last-minute conflicts such that four participants attended the English focus group on 1 June 2023 and eight the French focus group on 2 June 2023. IU-W and AB facilitated the English focus group, while AM and BAD facilitated the French focus group. The discussions were recorded, and the English transcribed by Otter.ai and the French by Amberscript. The French transcription was translated into English using automated translation software (DeepL) and revised by AM and BAD.

#### Data collection and analysis

The facilitation materials were developed in English following the input and deliberations from the workshop with TGLF and after several iterations among the research team. They were later translated into French using DeepL and validated by French-speakers on the study team. The session was divided into two parts: (i) why do practitioners choose to engage in the Movement, (ii) who makes what contribution [25]. For parts (i) and (ii), participants were asked to review the results from phase I and phase II and critically reflect on the sustainability implications for the IA2030 Movement. The semi-structured facilitation guide and probe questions were a product of iterations between the facilitators of both sessions (IU-W, AB, AM and BAD). The content of focus groups was systematically coded per a pre-established codebook (S1 Table). The analysis aimed to identify recurring reflections and interpretations. The findings are summarized and are incorporated in the presentation of the results in a narrative manner [28, 30].

### Ethical considerations

Ethical clearance was granted on 17 April 2023, by The Geneva Learning Foundation Commission on Research Ethics (number: 1.2023). Privacy was safeguarded by data management complying with the General Data Protection Regulation [37]. Consent was asked for participants in each research phase, and pseudo-anonymity assured. Data processing and reporting were anonymized. All participants were informed that participation was voluntary. All participants provided written consent to being recorded before the start of the workshop and focus group discussions.

### Inclusivity in global research

Additional information regarding the ethical, cultural, and scientific considerations specific to inclusivity in global research is included in the Supporting Information. (S1 Checklist).

## Results

### Respondent characteristics

Table 1 summarizes the demographic findings for the three surveys. Males accounted for 72% of responses to the Application Survey, 72% of the Impact Accelerator reports, and 79% of responses to the Sustainability Survey. Most of the applicants (98%) were from LMICs, with a high representation (53%) from countries in West Africa. Respondents to each survey included a diverse mix across health system level, profession and years of experience, with substantial participation (at least 12% of respondents) in each category in each survey (with the exception of “Other Health Professional” in the Application Survey).

**Table 1:** Baseline characteristics of respondents to the IA2030 Movement surveys.

Baseline Characteristics	Application (2022)		Impact Accelerator (2022)		Sustainability (2023)	
	N	%	N	%	N	%
**Gender**						
Male	4066	72%	296	72%	161	79%
Female	1616	28%	116	28%	44	21%
**Level of Health System**						
Health Facility	1034	19%	115	28%	49	24%
District	1308	25%	144	35%	74	36%
Region	1277	24%	87	21%	49	24%
National	1702	32%	66	16%	33	16%
**Profession**						
Other Health Professional	147	3%	48	12%	33	16%
Community Health Worker	1063	19%	57	14%	26	13%
Medical Doctor	1656	29%	116	28%	60	29%
Nurse	1017	18%	82	20%	30	15%
Public Health Specialist	1799	32%	109	26%	56	27%
**Years of Experience**						
Less than 3 years	1797	32%	68	17%	31	15%
3–5 years	1574	28%	87	21%	42	20%
6–10 years	1193	21%	103	25%	54	26%
11–16 and 16+ years	1118	20%	154	37%	78	38%
**Total**	**5682**	**100%**	**412**	**100%**	**205**	**100%**

### Composition of focus groups

The English-language focus group was composed of four IA2030 Movement members (one male, three females) from Kenya, Ghana and Nigeria whose experiences and backgrounds varied (public health specialist, public health anthropologist, researcher and disease control officer). The French-language focus group had eight members (four males and four females) from Mali, Madagascar, Guinea, Burkina Faso (n = 3), Morocco and Senegal. Participants were public health specialists (n = 4), a doctor, a nurse, a community health worker and a researcher. Participants worked from facility to national levels. The focus groups helped to interpret and extend quantitative findings and generate considerations for sustainability.

### Social and human capital: Engagement and motivation

#### Frequency

**Fig 1:**
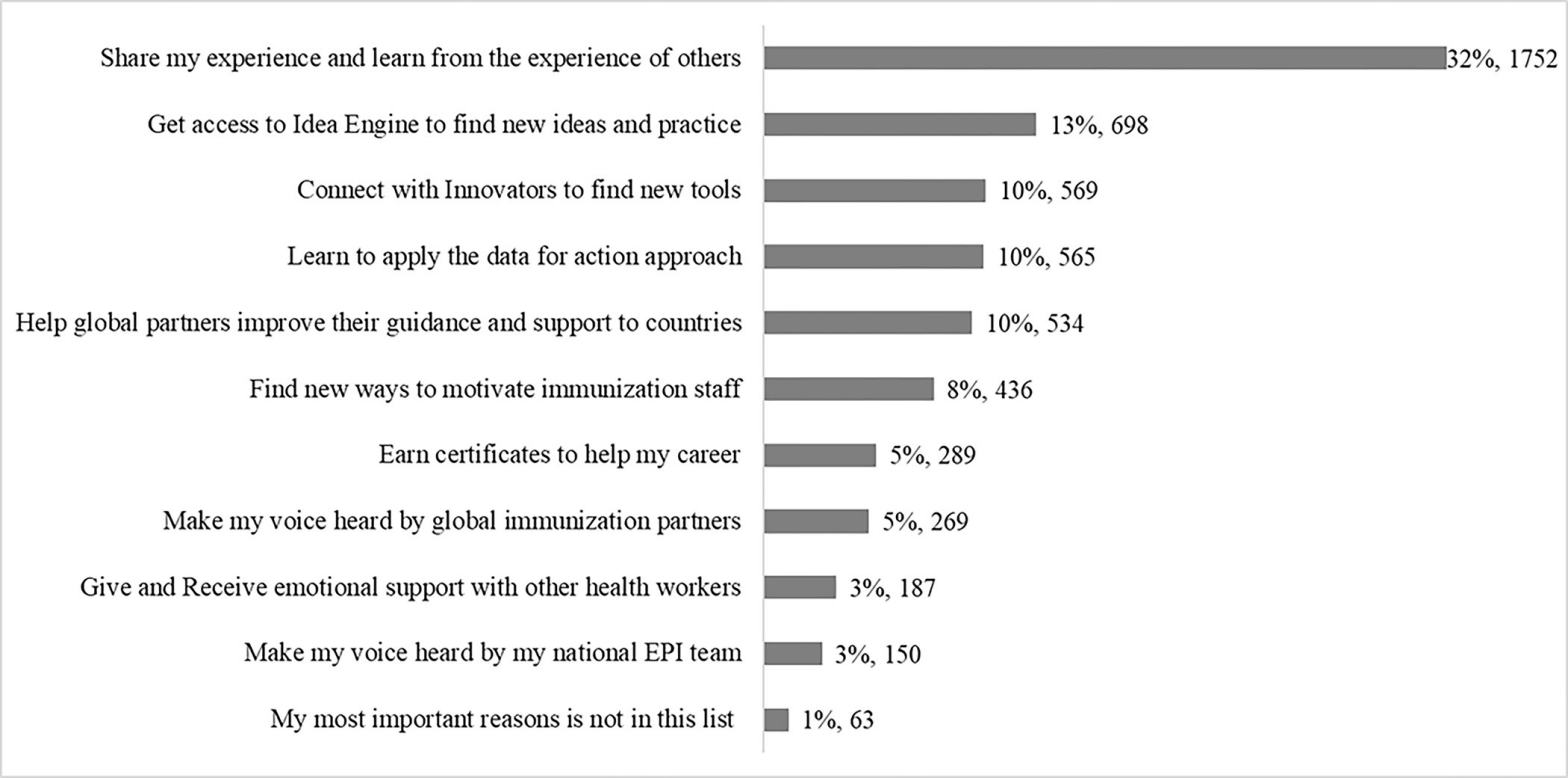
Responses (n = 5512) to the question ‘What is your most important reason to join the Movement for Immunization Agenda 2030?’. Source: Application Survey, IA2030 Movement (2022).

On the Application Survey, participants were asked about their reasons for joining the IA2030 Movement. The most common reason given, 2.46x the next highest response, was to share experiences and learn from the experiences of others (32%, n = 1752). The second most commonly given reason was getting access to the “Idea Engine” to find new ideas and practice (13%, n = 698). For the IA2030 Movement members who responded, earning certification was rarely an important reason for joining (5%, n = 289) (Fig 1).

**Fig 2:**
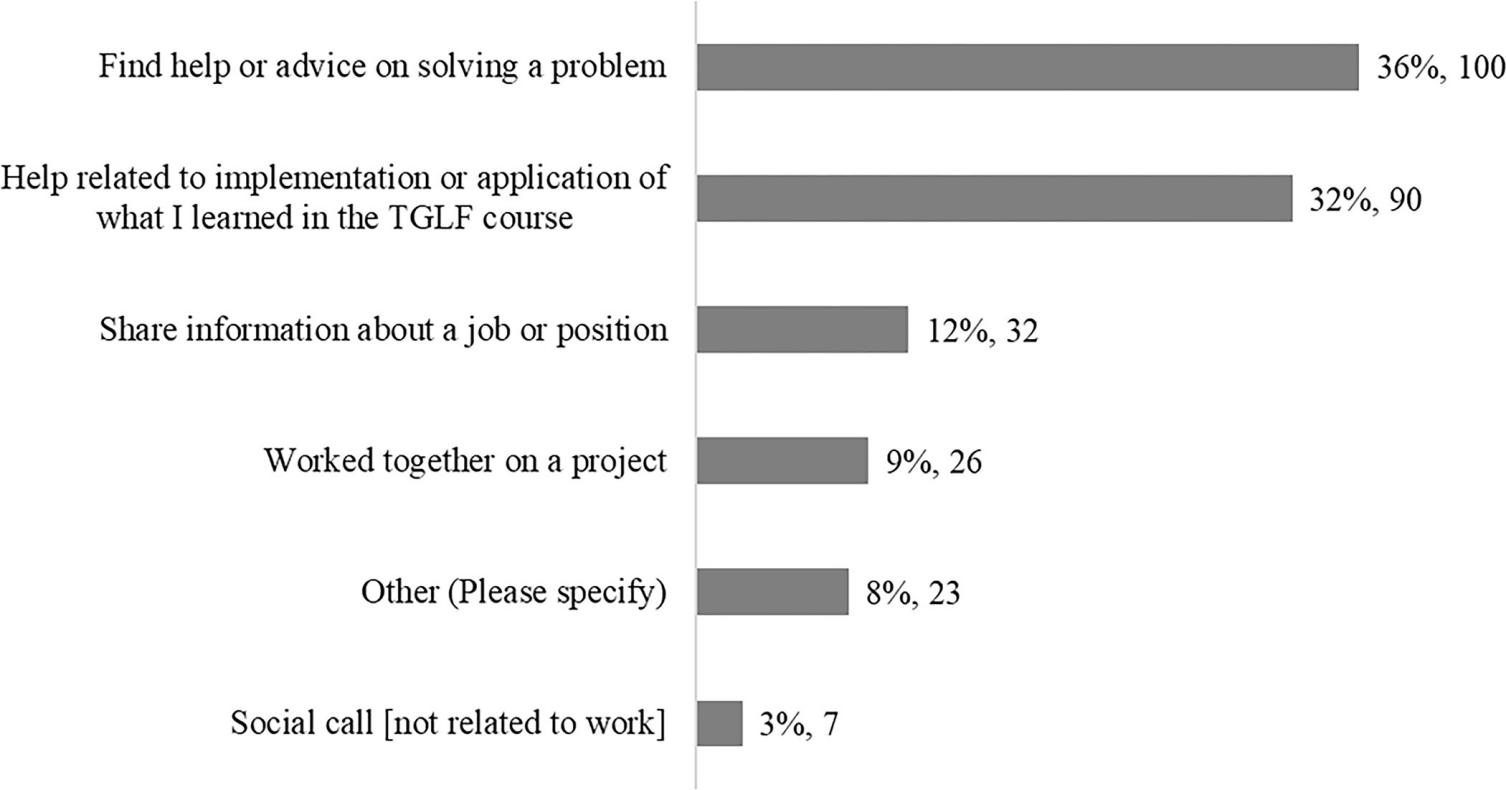
Responses (n = 278) to the question ‘If you have made contact with at least one other IA2030 Movement member, what was the main reason?’. Source: Sustainability Survey (2023).

The Sustainability Survey asked about the main reasons for interacting with peers. Most respondents (68%, n = 190) prioritized support for either applying what they are learning in the course or more generally seeking advice to solve a problem they face in their work context (Fig 2).

Focus group discussions were triangulated with responses to these two survey questions. Participants in both cohorts, French and English, affirmed that their engagement was driven by the wish to collaborate and contribute. An additional comment made was that mutual trust and respect are fundamental aspects of this engagement. Participants ascribed value to relying on each other, believing that peers are competent and reliable, and valuing the contributions that each member has to offer:

“*the aim of converging ideas and actions to improve practices and everything that’s being done for better vaccination coverage, for the well-being of our communities (…) also to learn from others who have carried out successful actions elsewhere that we’d like to know about to adapt it to our context*” [Speaker 4, Male, Medical Doctor, 11–15 years’ experience, French focus group].

#### Relationships

In order to better understand factors affecting practitioners’ rationale for joining the Movement, a logistic regression model from Key Influence visual of Power BI was used to examine the relationship between participants’ reason for joining and (i) demographic factors (gender, professional experience, health system level) and (ii) seven questions examining organizational learning culture [33]. S2 Table shows the odd ratios (95% confidence interval) of participant reasons for joining the IA2030 Movement relative to (i) demographic factors (gender, professional experience, health system level) and (ii) organizational learning culture (DLOQ). The complete visuals are displayed in S1 Fig.

The main reason given for joining the Movement, “To share my experience and to learn from the experience of others”, was particularly likely to be selected by those with high levels of experience and those working in an environment with a relatively high learning culture (DLOQ score). Practitioners in these environments were also more likely to select “To connect with innovators to find new tools and services”. This suggests that an organization’s learning culture is associated with a desire to seek out innovations and to learn from others.

The desire “To help global partners improve their guidance and support to countries” and “To make my voice heard by global partners” was a particularly strong rationale for those with low levels of experience, those working in a health facility and women. This suggests an interest among those at early stages of their career and working at the frontline to feed into global guidance and ensure its relevance to everyday immunization activities.

Being a woman was associated with three distinct rationales: “Make my voice heard by global partners”, “Help global partners improve their guidance and support to countries”, and “Get access to Idea Engine to find new ideas and practice”. A female French focus group participant explained:

“*I got involved in the movement, first of all to make my voice heard, because there were a lot of things, I was sure of. The minister, perhaps, isn’t aware, (…) of what’s going on in the healthcare facilities. There’s also the sharing of experience (…) the fact that I’m developing my skills a bit [and] (…) I know what’s going on in other countries too, by sharing my experience so that I can move forward in this field.”* [Speaker 5, Female, Community Health Worker, 6–10 years’ experience, French focus group]

Focus group participants expressed the view that sharing experiences and providing support to each other were the most important reasons to join the Movement and were perceived as the most valuable aspect of this network:

“*Obviously, joining the movement improves our knowledge and skills, but it also and above all enables us to share and receive good practices and experiences from others*.” [Speaker 2, Male, Clinical Research, 6–10 years’ experience, French focus group]

### Financial capital: Contributions and sustainability

To generate insights into how the IA2030 Movement might be sustained, the Sustainability Survey assessed levels of participant engagement and investment in the Movement, in terms of their time and other resources. Donors contributed to TGLF’s costs to develop and then support the Movement during the Full Learning Cycle in 2022, building on TGLF’s six-year investment in developing its pedagogical and digital infrastructure. This meant that these facilitation costs did not have to be covered by participants: there were no “fees” for participants or their employers, but also no “per diem” or other financial compensation was offered, even though such financial incentives are considered a requirement in many immunization capacity-building activities[38]. Participants therefore had to find solutions to fund their own connectivity, technology and make decisions with respect to the opportunity cost of participating.

We found that 87% (n = 253) of respondents reported engaging in the peer learning activities (such as online learning sessions) at their own cost and without financial compensation (salary or *per diem*) although these activities were often held during working hours in most countries and they were encouraged to embed activities as part of their daily paid work (if they reported that their supervisor did not agree with their participation they were not accepted into the Movement and Full Learning Cycle). 97% (n = 281) used their personal electronic devices such as computer and mobile phone to participate. These findings indicate that participants saw high enough value in participating and that it was worth their personal investment. This could also be interpreted as further indication of high levels of intrinsic motivation, as suggested by earlier studies on Movement members (see S2 Table).

Focus group participants reported regular use of personal resources to take part in Movement activities.

“*(…) almost all the time, I use my personal data for [the] four years of commitment (…) I use my personal data. My district provides support. [My boss] allows me to have that space (…) [He] does not bother me with office duties when I join movement session or anything concerning the movement.*” [Speaker 4, Male, Disease Control, 6–10 years’ experience, English focus group].

Some focus group participants raised concerns about the time commitment and financial cost of Movement activities, although each participant chose which sets of activities to join, and many have sustained such engagement over several years prior to the IA2030 Movement (exemplified in the quote above).

“*It was extremely hard for me to like cope with all of the timing, you know, the timeline of submissions, deadlines and all that. So I will see as going forward, it would be really hard for people to like, really sit and like even those numbers of hours that most of us have given before. Because most times (…) it’s part of the work hours that we’re using to come up with this action plans, even though it’s not part of our various workspaces*.” [Speaker 2, Female, Public Health, 6–10 years’ experience, English focus group]

As part of the Full Learning Cycle, each participant was invited to develop a tailored action plan to address an immunization challenge in their local context. They were encouraged to draw on the Ideas Engine and situational analysis if they had participated in either or both of those preceding parts of the Full Learning Cycle. By the end of the first month of implementation in 2022, almost one-third (32%, n = 70) reported having completed implementation of their action plans, while others anticipated longer implementation periods. Most participants (77%, 209) stated that action plan implementation was done as part of their daily work. Members dedicated a median of 6 hours per week (n = 211) to action plan implementation and reported that they and/or their organization spent a median of 200 USD on implementation.

When asked about funding for action plan implementation, 47% (n = 136) answered that they paid personally out of pocket for implementation, 33% (n = 96) stated that no funding was necessary, and 8% (n = 22) that their employers funded implementation of their action plan. The percentage who reported self-funding implementation was lower than those who reported self-funding participation in peer-learning activities, but is still surprisingly high given that implementation was generally done as part of their employment (i.e. daily work).

Across the two focus groups, narratives appeared to be congruent with the survey findings. These findings suggest that some practitioners are choosing to subsidize implementation of their action plans as part of their commitment to their work responsibilities (**Table 2**). A quote from the FGs further illustrates this:

“*I have spent a whole lot on implementing or coming up with action plans and the time that I have used in terms of…resources, like data bundles, and all that. Sometimes I have to…subscribe to different networks for sustainability…one network would drop; you’d have to reconnect with another one. So it’s like, different bundles for different networks, just to keep up with the work here*.” [Speaker 2, Female, Public Health, 6–10 years’ experience, English focus group]

**Table 2:** Summary of sustainability survey.

IA2030 Movement activities				
**Does someone provide you a salary when you are participating in IA2030 Movement activities? For example, you are supported by your employer to participate during your work hours**	**Number**	**%**		
No	253	87%		
Yes	38	13%		
**Paid out of pocket for data during the Movement**	**Number**	**%**		
No	10	3%		
Yes, all activities	281	97%		
**Hours per week spent on Movement activities**	**Median**	**Mean**	**Std. dev.**	
Expected	7	11.43	15.96	
Spent	6	11.53	29.19	
**Spending each month on communications (USD)**	**Median**	**Mean**	**Std. dev.**	
	25.0	56.6	80.1	
**Action Plan**				
**Implementation of action plan as part of employment**	**Number**	**%**		
No	61	23%		
Yes	209	77%		
**Someone provides funding towards the implementation of your action plan**	**Number**	**%**		
No funding was necessary to implement my action plan	96	33%		
Yes, another organization	14	5%		
Yes, I paid personally out of pocket	136	47%		
Yes, my colleagues and I shared the costs	15	5%		
Yes, my employer	22	8%		
Yes, the community	8	3%		
**Funding used for implementation in USD**	**Median**	**Mean**	**Std. dev.**	
	200	777	1,471	
**Hours per week spent implementing the action plan**	**Median**	**Mean**	**Std. dev.**	
	6	10.17	14.98	

**Fig 3:**
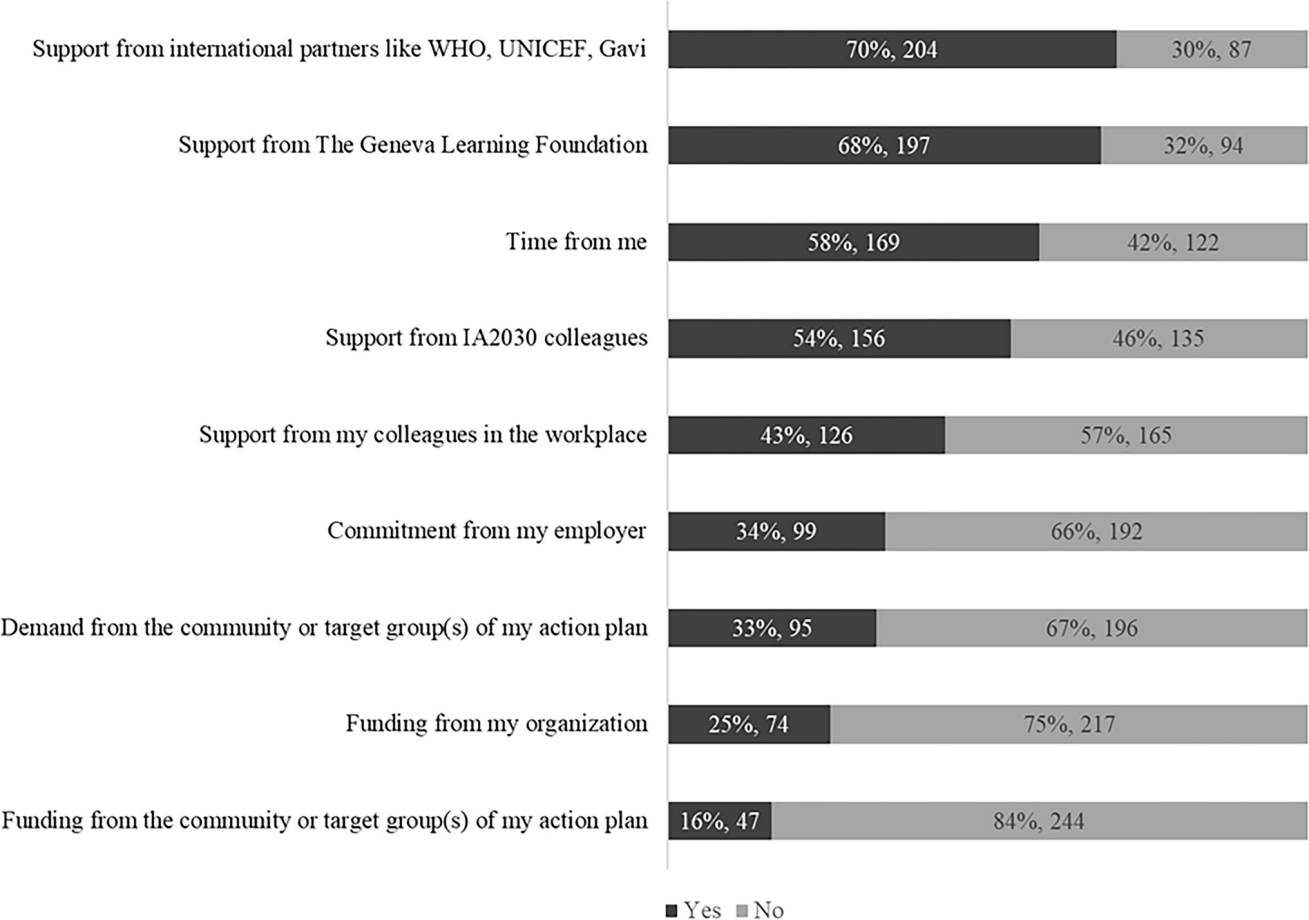
What would be most helpful to sustain your commitment to achieving the goals of IA2030? N = 291. Source: Sustainability Survey (2023). Respondents could select more than one answer.

The Sustainability Survey also asked Movement members what they would find most helpful to sustain their commitment to achieving the goals of IA2030 through local action. The most favored response was support from international partners such as WHO, UNICEF and Gavi (70%, 204) and TGLF (68%, n = 197). By contrast, only 16% (n = 47) of participants responded that funding from the community would be the most helpful towards sustainability, and 25% (n = 74) selected funding from their own organization (Fig 3).

In the focus groups, some participants further elaborated that support was not limited to funding, but also in terms of visibility and recognition of their IA2030 activities and commitment:

“*the focus isn’t on bringing in additional resources from the community or from a, you know, target group of some sort, or from your own organization. But I do very much take that to heart…that what may be needed from individual organizations could be visibility, or connections or support as opposed to specific funding”* [Speaker 1, Female, Nurse, 16+ years’ experience, English focus group].

## Discussion

This study investigated the reasons why health practitioners in LMICs, particularly in Africa, choose to participate in the IA2030 Movement, the resource requirements and sources to support them in so doing, and considerations for sustainability for the Movement over the longer term. It provides critical insights that can inform other digital networks of local actors working toward local and global goals in LMICs. In summary, we found high levels of individual commitment to networking and learning from peers in order to achieve immunization goals. Quantitative survey findings were corroborated by focus group discussions.

Similar to other reports, this study found that the **reasons health practitioners chose to engage** vary, with sharing experiences and learning from others the most common rationale [24, 39]. Reasons for joining varied across gender, profession, years of experience and level within the health system.

Regardless of the specific reason for joining, a digital peer-learning network creates the flexibility for a diverse range of practitioners to find the value they need to engage. The focus group discussion was consistent with the premise that a strong foundation for the Movement is mutual trust, where participants expressed a shared belief and confidence among individuals involved in the collective action initiative. This includes feeling comfortable depending on each other, believing that everyone is competent and reliable, and valuing the contributions that each member has to offer.

The **scale of commitment and investment** that each member chooses to make helps to quantify the individual value each places on being part of the Movement. Although activities are not year-round, during Full Learning Cycles the member commitment can be estimated at an average of 6 hours/week, generally without supplementary salary or other extrinsic motivation, with a median of US$25/month spent on connectivity costs. This is a significant investment. Furthermore, participants are taking emergent learnings into their regular work by implementing locally tailored action plans as part of their employment at a median cost of US$200 and funded by resources health workers identified locally. There is little or any comparable data on personal investments made by health workers in LMICs to such an initiative, but a rapidly growing set of first-person case studies emphasize the high levels of personal commitment being made by Movement members [19–22].

Shared goals and mutual trust are important constructs of social and human capital [26, 29]. Studies that investigated environmental collective actions pointed that those two are intrinsically connected to cooperation building and can evoke change [40–42]. They argue that with financial capital and access and communication with external sources, collective action would further strengthen its pivotal role in promoting change [40–42]. Health workers motivated to improve services by investing in their own learning and connecting with peers are also often constrained by context, such as coming from resource-limited settings. Therefore, as highlighted by focus group participants, the success of the IA2030 Movement depends on both collective action by individuals and support of organizations such as TGLF that facilitate it [29].

Other digital learning initiatives [10, 13] in global health have demonstrated that such platforms or networks can function at scale, transfer knowledge on a diversity of topics, and to a lesser extent actively support peer learning. However the investment made by participants to sustain learning networks, including how participants support peers, and how participants turn knowledge into local action and evidence of the associated impact, remains under-researched and under-quantified by digital learning initiatives.

Findings from the research highlight two intertwined implications for the IA2030 movement. First, the study demonstrates that shared goals, mutual trust, and personal dedication provide a solid foundation for the Movement, highlighting the critical role of social and human capital. The intrinsic motivation to engage is promising, yet it is too early to determine if the level of individual commitment will be sustainable in the long term. This leads to the second implication. Movement members are substantial “investors” in the Movement and achieving the goals of IA2030. For the Movement to endure and grow, complementary but relatively modest commitments from a network of funders or participant’s employers into organizations that maintain the digital infrastructure and facilitate the peer learning approaches are also necessary. Strengthening the networks and supporting local health workers to lead local change will be crucial to sustaining collective action and achieving national and global immunization targets.

## Limitations

Data are self-reported. The application survey results are a product of the participants’ application process. There might be a potential bias towards providing answers that align with what they believed would enhance their chances of being accepted into the program, which could influence the accuracy and authenticity of the data. The sustainability survey’s response rate was 29%, which could introduce potential response bias, as those who chose to respond may have different characteristics or motivations than non-respondents. Time and cost estimates from respondents were not independently verified, although focus group participants were asked if aggregated estimates (e.g. mean, median) seemed reasonable. It was not possible to identify comparative data for a number of data points, such as the extent to which health workers in LMICs fund their own learning, or what portion is funded by their governments and partners. In that respect, this study may provide a useful starting point for future studies to build upon. The contributions of TGLF to the network, and associated value, could also be explored in further research.

The research depended on the willingness of health practitioners to contribute to it. Despite the high response rate to the sustainability survey, with almost three-quarters expressing a willingness to participate in focus group discussions, participation in the focus groups was lower than anticipated. Survey and focus group participants may represent an exceptionally motivated or engaged sample. If so, their insights may not be representative of the whole. However, this does not diminish their relevance or importance: sustainability approaches that do not respond to or reflect the interests of the motivated may be even less likely to address less-engaged colleagues.

International development organizations are a critical part of the global health ecosystem. Their contribution to the Movement in 2022 and 2023 has been relatively limited in comparison to the scale of contributions from Movement members documented in this paper. Therefore, the research in this paper necessarily focused primarily on members and their interactions with TGLF.

The transdisciplinary methods of this study offers both strengths and limitations. It enriched the research, knowledge exchange and potential for impact and sustainability by prioritizing diverse data from LMIC health workers [28, 43]. However, a challenge for interpretation is balancing the scale of quantitative data points relative to the qualitative data. Additionally, as the transdisciplinary research methods were tailored to LMIC Movement members, they limited the data from international development organizations within the study.

## Conclusions

Long-standing development challenges are calling for increasingly localized solutions to deliver results. The Movement for IA2030 is already demonstrating that collective action has the potential to scale across LMICs and types of health practitioners, building solutions from communities up. If the Movement can be sustained, it has a very real potential to impact the trajectory to achieving IA2030 goals.

Sustaining the Movement will implicate a diverse cross-section of stakeholders, such as:

1. Local and national stakeholders and Movement members that could continue to define and articulate the value they want from digital peer learning networks, whether that be supporting local and national action, supporting peers in other countries, or advocating to international organizations and funders.
2. International development organization and funders that could increasingly incorporate knowledge generated and local action catalyzed by decentralized networks, which may lead them to rethink what and how they invest in global health programs.
3. Policy makers that could urgently value and commit to collective action and experiential knowledge for addressing tenacious health problems in challenging LMIC contexts.
4. Researchers that could strengthen the understanding of collective action and intrinsic motivation in LMICs for addressing health and development challenges through digital peer learning networks, including the Movement for IA2030.

For other learning networks, these findings suggest that digital approaches offer the possibility not only for increased scaling of locally tailored actions to contribute to solving global challenges, but also for increased efficiency and the potential to contribute to offsetting generations of inequities and hierarchies that underpin development support. Networks that prioritize peer-learning, local action, and knowledge sharing have the potential to drive significant change in diverse sectors, from healthcare to environmental sustainability to education and beyond. Therefore, investing in and sustaining such networks could be critical tools for addressing both local and global challenges. Any one of these possibilities seems justification for pursuing digital peer-learning networks to their fullest potential.

Yet the journey is just beginning as we seek to understand how the digital transformation of peer learning from local (“I learn from my supervisor, or colleagues and community I work with”) to networked (“I learn from everyone, everywhere, at any time”) will evolve and impact global development. This paper helps to document the attractions of this approach from the perspective of health workers across LMICs. An accompanying paper and case studies are beginning to further capture insights into impacts.

## Supporting information

S1 Checklist Inclusivity in global research. (DOCX)

S1 Table Code book for focus group thematic analysis. (DOCX)

S2 Table Odd ratios (95% confidence interval) for a participants’ reasons for joining the IA2030 Movement relative to (i) demographic factors (gender, professional experience, health system level) and (ii) organizational learning culture (DLOQ) (n = 5512). (DOCX)

S1 Fig Power BI key influencer outcome. (TIF)

## References

[1] WHO. Immunization Agenda 2030: A Global Strategy to Leave No One Behind [Internet]. 2023. Available from: https://www.who.int/teams/immunization-vaccines-and-biologicals/strategies/ia2030 10.1016/j.vaccine.2022.11.042 39004466

[2] WHO. Regaining Lost ground: IA2030 partnership Progress report 2022 [Internet]. Geneva; 2023 [cited 2023 Jun 7] p. 26. Available from: https://www.immunizationagenda2030.org/ia2030-annual-reports

[3] Lindstrand A, Cherian T, Chang-Blanc D, Feikin D, O’Brien KL. The World of Immunization: Achievements, Challenges, and Strategic Vision for the Next Decade. J Infect Dis. 2021 Sep 30;224(Supplement_4):S452–67. 34590130 10.1093/infdis/jiab284 PMC8482029

[4] Decouttere C, De Boeck K, Vandaele N. Advancing sustainable development goals through immunization: a literature review. Glob Health. 2021 Aug 26;17(1):95. doi: 10.1186/s12992-021-00745-w 34446050 PMC8390056

[5] WHO. Seventy-fourth World Health Assembly [Internet]. 2021 p. 7. Report No.: A74/9 Add.4. Available from: https://apps.who.int/gb/ebwha/pdf_files/WHA74/A74_9Add4-en.pdf

[6] Immunization Scholar Pledge for Impact [Internet]. The Geneva Learning Foundation. [cited 2023 Jul 18]. Available from: https://www.learning.foundation/immunization-agenda-2030-year1

[7] Dewey John. Dewey J. Experience and education. In: The educational forum. Taylor & Francis; 1986. p. 241–52. In: The educational forum [Internet]. Taylor & Francis; 1986. p. 241–252. Available from: 10.1080/00131728609335764

[8] Spero Ken. Scenario-based E-learning. American Society for Training and Development; 2012.

[9] Lee Edwin LS, Lee Wing On. Enabling Continuous Innovation and Knowledge Creation in Organizations: Optimizing Informal Learning and Tacit Knowledge. In: Third International Handbook of Lifelong Learning [Internet]. Springer; 2023. Available from: https://link.springer.com/10.1007/978-3-030-67930-9_68-1

[10] Utunen H, Mattar L, Piroux C, Ndiaye N, Christen P, Attias M. Superusers of Self-Paced Online Learning on OpenWHO. In: Mantas J, Gallos P, Zoulias E, Hasman A, Househ MS, Diomidous M, et al., editors. Studies in Health Technology and Informatics [Internet]. IOS Press; 2022 [cited 2024 Sep 14]. Available from: https://ebooks.iospress.nl/doi/10.3233/SHTI220648 10.3233/SHTI220648 35773794

[11] Eller K. Learning to (Co)Evolve: A Conceptual Review and Typology of Network Design in Global Health Virtual Communities of Practice. Interdiscip J Inf Knowl Manag. 2024;19:021.

[12] Dagenais C, Proulx M, McSween-Cadieux E. Using Digital Platforms in Schools for Prevention and Health Promotion: A Scoping Review. Health Behav Policy Rev [Internet]. 2022 Mar [cited 2024 Sep 7];9(2). Available from: https://www.ingentaconnect.com/contentone/psp/hbpr/2022/00000009/00000002/art00001

[13] Hunt, Richard C, Struminger, Bruce B, Redd, John T, Jolly, B Tilman, B Tilman, Arora, Sanjeev, et al. Virtual peer-to-peer learning to enhance and accelerate the health system response to COVID-19: the HHS ASPR Project ECHO COVID-19 Clinical Rounds Initiative. Annals of Emergency Medicine. 2021;78(2):223–228. doi: 10.1016/j.annemergmed.2021.03.035 34325856 PMC8052469

[14] Sabin Vaccine Institute. Boost Community [Internet]. Boost Community. Available from: https://boostcommunity.org/

[15] Kalantzis Mary, Cope Bill. New learning: elements of a science of education. Second edition. Cambridge University Press; 2012.

[16] Marsick VJ, Watkins KE, Scully-Russ E, Nicolaides A. Rethinking informal and incidental learning in terms of complexity and the social context. J Adult Learn Knowl Innov. 2017 Dec;1(1):27–34.

[17] Siemens George. Connectivism: A Learning Theory for the Digital Age [Internet]. 2005 [cited 2023 Jul 18]. Available from: http://www.itdl.org/Journal/Jan_05/article01.htm

[18] Marsick VJ, Watkins KE. Informal and Incidental Learning. New Dir Adult Contin Educ. 2001;2001(89):25.

[19] Jones Ian, Sadki Reda, Brooks Alan, Gasse François, Mbuh Charlotte, Zha Min, et al. IA2030 Year 1 report. Consultative engagement through a digitally enabled peer learning platform [Internet]. Zenodo; 2022 Sep [cited 2023 Mar 19]. Available from: 10.5281/zenodo.7119648

[20] Monzón, María Fernanda, Jones, Ian, Mbuh, Charlotte, Sadki, Reda. IA2030 Case study 17. María Fernanda Monzón. Speaking up for frontline staff [Internet]. Report No.: 17. Available from: 10.5281/zenodo.7785024

[21] Faye, Wasnam, Jones, Ian, Mbuh, Charlotte, Sadki, Reda. IA2030 Case study 18. Wasnam Faye. Vaccine angels—Give us the opportunity and we can perform miracles. [Internet]. Report No.: 18. Available from: 10.5281/zenodo.7785244

[22] Monga, Franck, Jones, Ian, Mbuh, Charlotte, Sadki, Reda. IA2030 Case study 19. Franck Monga. Building a movement for IA2030 in the Democratic Republic of Congo [Internet]. Available from: 10.5281/zenodo.7794726

[23] Watkins KE, Marsick VJ. Informal and Incidental Learning in the time of COVID-19. Adv Dev Hum Resour. 2021 Feb;23(1):88–96.

[24] Watkins KE, Sandmann LR, Dailey CA, Li B, Yang SE, Galen RS, et al. Accelerating problem-solving capacities of sub-national public health professionals: an evaluation of a digital immunization training intervention. BMC Health Serv Res. 2022 Dec;22(1):736. doi: 10.1186/s12913-022-08138-4 35655276 PMC9161754

[25] Ostrom E. Analyzing collective action: Analyzing collective action. Agric Econ. 2010 Nov;41:155–66.

[26] Rycroft-Malone J, Burton CR, Wilkinson J, Harvey G, McCormack B, Baker R, et al. Collective action for implementation: a realist evaluation of organisational collaboration in healthcare. Implement Sci. 2015 Dec;11(1):17. 10.1186/s13012-016-0380-z PMC4748518 26860631

[27] Creswell JW, Creswell JD. Research design: Qualitative, quantitative, and mixed methods approaches. Sage publications; 2017.

[28] Leavy P. Essentials of transdisciplinary research: Using problem-centered methodologies. Routledge; 2016.

[29] Rahman HMT, Hickey GM, Sarker SK. A framework for evaluating collective action and informal institutional dynamics under a resource management policy of decentralization. Ecol Econ. 2012 Nov;83:32–41.

[30] Steinmetz-Wood M, Pluye P, Ross NA. The planning and reporting of mixed methods studies on the built environment and health. Prev Med. 2019 Sep;126:105752. doi: 10.1016/j.ypmed.2019.105752 31226344

[31] Sadki Reda, Mbuh Charlotte, Zha Min, Gasse François, Brooks Alan. Localizing Commitments, Challenges, and Insights on the Road to Immunization Agenda 2030: Responses from 6,185 national and sub-national staff (Immunization Agenda 2030 Full Learning Cycle, 7 March—20 June 2022). 2023.

[32] Schaufeli WB, Bakker AB, Salanova M. The Measurement of Work Engagement With a Short Questionnaire: A Cross-National Study. Educ Psychol Meas. 2006;66(4):701–16.

[33] Watkins KE O’Neil J. The Dimensions of the Learning Organization Questionnaire (the DLOQ): A Nontechnical Manual. Adv Dev Hum Resour. 2013 May;15(2):133–47.

[34] Guay Frédéric, Vallerand Robert J., Blanchard Céline. On the assessment of situational intrinsic and extrinsic motivation: The Situational Motivation Scale (SIMS). Springer. 2000;24:175–213.

[35] Sufi FK. AI-GlobalEvents: A Software for analyzing, identifying and explaining global events with Artificial Intelligence. Softw Impacts. 2022 Feb 1;11:100218.

[36] Sufi FK. AI-Landslide: Software for acquiring hidden insights from global landslide data using Artificial Intelligence. Softw Impacts. 2021 Nov 1;10:100177.

[37] Union European. Regulation (EU) 2016/679 of the European Parliament and of the Council. 2016/679 2016.

[38] Mitchell V. Framework for Immunization Training and Learning [Internet]. Seattle, USA: The Bill and Melinda Gates Foundation; 2017. Available from: https://www.nesi.be/wp-content/uploads/2020/04/Framework-for-Immunization-Training-and-Learning_distribution.pdf

[39] Jones I, Watkins KE, Sadki R, Brooks A, Gasse F, Yagnik A, et al. IA2030 Case Study 7. Motivation, learning culture and programme performance [Internet]. Zenodo; 2022 Aug [cited 2023 Jul 2]. Available from: 10.5281/zenodo.7004304

[40] Rahman HMT, Hickey GM, Sarker SK. Examining the Role of Social Capital in Community Collective Action for Sustainable Wetland Fisheries in Bangladesh. Wetlands. 2015 Jun;35(3):487–99.

[41] Adhikari B, Di Falco S. Social Inequality, Local Leadership and Collective Action: An Empirical Study of Forest Commons. Eur J Dev Res. 2009 Apr;21(2):179–94.

[42] Adger WN. Social Capital, Collective Action, and Adaptation to Climate Change. Econ Geogr. 2009 Feb 16;79(4):387–404.

[43] Kok MO, Gyapong JO, Wolffers I, Ofori-Adjei D, Ruitenberg J. Which health research gets used and why? An empirical analysis of 30 cases. Health Res Policy Syst. 2016 Dec;14(1):36. doi: 10.1186/s12961-016-0107-2 27188305 PMC4869365

