# Supplementary material for: Towards a sustainable model for a digital learning network in support of the Immunization Agenda 2030 –a mixed methods study with a transdisciplinary component
Source: PLOS Glob Public Health. 2024 Dec 31;4(12):e0003855. doi: 10.1371/journal.pgph.0003855 (PMC11687746; doi:10.1371/journal.pgph.0003855)
Supplement: S1 Table — (DOCX) [file pgph.0003855.s002.docx]

**S1 Table Code Book for Focus Group Thematic Analysis**

|  | Themes and Sub-themes | Definition | Illustration Quotes |
| --- | --- | --- | --- |
| **Engagement** | 1. Collaboration and Cooperation: | their ability to work effectively in teams, engage in cooperative decision-making, share resources, and actively contribute to collaborative efforts. | “the aim of converging ideas and actions to improve practices and everything that's being done for better vaccination coverage, for the well-being of our communities” (Speaker 4) |
|  | 1. Mutual trust and respect | refers to the shared belief and confidence among individuals or groups involved in the collective action initiative. Including, feeling comfortable depending on each other, believing that everyone is competent and reliable and valuing the contributions that each member has to offers. | “also to learn from others who have carried out successful actions elsewhere that we'd like to know about to adapt it to our context” (Speaker 4) |
|  | 1. Perceived Impact | participants' beliefs, opinions, and judgments about the extent to which the collective action has achieved its intended goals or made a positive difference in addressing immunization | “year after year, to monitor the group's progress” (Speaker 2) |
|  | 1. Satisfaction levels | participants' subjective feelings of contentment, fulfillment, or gratification with their involvement and the outcomes of the collective action initiative. | “The various teaching meetings, i.e., the training courses that this movement provides, also constitute an added value that enables us to better fine-tune our work in the field, taking into account several aspects, be it communication or the provision of services” (Speaker 6) |
| **Contribution** | 1. Commitment/   resources | includes tracking the number of hours contributed, financial contributions, or the effort put into collective action activities. | “community relay helped us a lot, especially to reach the hard-to-reach areas since they were on the ground and also on the incentive side in relation to transportation costs and or fuel to go to these areas” (Speaker 2)  “We're not getting paid because we won't have paid for my participation in this. But as for the implementation of the action plan, it's a bit mixed. Not only because of GPG's personal contributions, but also through the communities and other players involved.” (Speaker 4) |
|  | 1. Advocacy and Outreach | individuals' efforts to raise awareness, mobilize support, or engage with external stakeholders. This includes activities such as organizing events, giving presentations, writing articles or blog posts, conducting outreach campaigns, or engaging in lobbying or advocacy work. | “(…)people who are here to learn and to share this information in the field to improve practices” (Speaker 2) |
|  | 1. Skills and Expertise | specific knowledge, qualifications, or specialized skills that individuals or groups contribute to the collective action efforts. | “We have researchers in the movement, communicators. You've got people who actually run vaccination sessions in the community. We also have partners who can be people, teams that mobilize financial resources, allocate funds. So everyone, at ~~their~~ **his** own level, can contribute in ~~their~~ **his** own way to the success of vaccination.” (Speaker 2) |
|  | 1. Leadership | individuals' involvement in leadership roles and decision-making processes. | “We can't live without the Foundation, nor can the Foundation without the members” (Speaker 6) |
|  | 1. Sustainability | the ability of a collective action initiative to maintain its effectiveness, impact, and relevance over the long term. | “help us the most to maintain the commitment to achieve the goals. I think most of the elements here are bound to come together. First of all, there's community commitment, whatever the contribution of international organizations and international partners. We also need a grassroots commitment to sustainability, as well as input from the departments we work for, and ongoing support from the movement and its colleagues, because there will always be things developed elsewhere that will also be of use to us” (Speaker 4) |
