# Supplementary material for: Towards a sustainable model for a digital learning network in support of the Immunization Agenda 2030 –a mixed methods study with a transdisciplinary component
Source: PLOS Glob Public Health. 2024 Dec 31;4(12):e0003855. doi: 10.1371/journal.pgph.0003855 (PMC11687746; doi:10.1371/journal.pgph.0003855)
Supplement: S2 Table — (DOCX) [file pgph.0003855.s003.docx]

**S2 Table Odd ratios (95% confidence interval) of participant reasons for joining the IA2030 Movement relative to (i) demographic factors (gender, professional experience, health system level) and (ii) organizational learning culture (DLOQ) (n= 5512)**

| **Reason to join: Share my experience and learn from the experience of others** | | | | | | | | | |
| --- | --- | --- | --- | --- | --- | --- | --- | --- | --- |
| *When Characteristics is* |  | *The Odds Ratio of reason to join* | | |  | | |  |  |
| Experience is 16 years + |  | 1.32x | |  | | |  |  |  |
| DLOQ > 4.1 (Low) |  | 1.31x | |  | | |  |  |  |
| Experience is 11 and 15 years |  | 1.2x | |  | | |  |  |  |
| Gender is man |  | 1.16x | |  | | |  |  |  |
| System level is District |  | 1.1x | |  | | |  |  |  |
| **Reason to join: Get access to the Ideas Engine to find new ideas and practices** | | | | | | | | | |
| *When Characteristics is* |  | *The Odds Ratio of reason to join* | | |  | | |  |  |
| DLOQ <5.4 (Very High) |  | 1.32x | |  | | |  |  |  |
| Gender is woman |  | 1.21x | |  | | |  |  |  |
| **Reason to join: Connect with innovators to find new tools and services** | | | | | | | | | |
| *When Characteristics is* |  | *The Odds Ratio of reason to join* | | |  | | |  |  |
| DLOQ >5.8 (Very High) |  | 1.45x | |  | | |  |  |  |
| System level is region |  | 1.36x | |  | | |  |  |  |
| **Reason to join: Learn to apply the “data for action” approach** | | | | | | | | | |
| *When Characteristics is* |  | *The Odds Ratio of reason to join* | | |  | | |  |  |
| DLOQ <5.1 (Very High) |  | 1.32x | |  | | |  |  |  |
| System level is region |  | 1.21x | |  | | |  |  |  |
| **Reason to join: Help global partners improve their guidance and support to countries** | | | | | | | | | |
| *When Characteristics is* |  | *The Odds Ratio of reason to join* | | |  | | |  |  |
| Experience is no experience |  | 1.52x | |  | | |  |  |  |
| System level is health facility |  | 1.42x | |  | | |  |  |  |
| Gender is women |  | 1.26x | |  | | |  |  |  |
| Experience < 3 years |  | 1.22x | |  | | |  |  |  |
| **Reason to join: Find new ways to motivate immunization staff** | | | | | | | | | |
| *When Characteristics is* |  | *The Odds Ratio of reason to join* | | |  | | |  |  |
| System level is district |  | 1.35x | |  | | |  |  |  |
| **Reason to join: Earn certificates to help my career** | | | | | | | | | |
| *When Characteristics is* |  | *The Odds Ratio of reason to join* | | |  | | |  |  |
| System level is health facility |  | 1.65x | |  | | |  |  |  |
| Experience is no experience |  | 1.58x | |  | | |  |  |  |
| Gender is man |  | 1.51x | |  | | |  |  |  |
| **Reason to join: Make my voice heard by global immunization partners** | | | | | | | | | |
| *When Characteristics is* |  | *The Odds Ratio of reason to join* | | |  | | |  |  |
| System level is health facility |  | 1.70x | |  | | |  |  |  |
| Experience is 3-5 years |  | 1.43x | |  | | |  |  |  |
| Gender is women |  | 1.33x | |  | | |  |  |  |
| **Reason to join: Give and receive emotional support with other health workers** | | | | | | | | | |
| *When Characteristics is* |  | *The Odds Ratio of reason to join* | | |  | | |  |  |
| Experience is no experience |  | 2.08x | |  | | |  |  |  |
| DLOQ >5.8 (Very High) |  | 1.79x | |  | | |  |  |  |
| **Reason to join: Make my voice heard by my national EPI team** | | | | | | | | | |
| *When Characteristics is* |  | *The Odds Ratio of reason to join* | | |  | | |  |  |
| Experience is 3-5 years |  | 1.49x | |  | | |  |  |  |
|  | | |  | | |  | | |  |
