# Supplementary figures and images for: Towards a sustainable model for a digital learning network in support of the Immunization Agenda 2030 –a mixed methods study with a transdisciplinary component
Source: PLOS Glob Public Health. 2024 Dec 31;4(12):e0003855. doi: 10.1371/journal.pgph.0003855 (PMC11687746; doi:10.1371/journal.pgph.0003855)

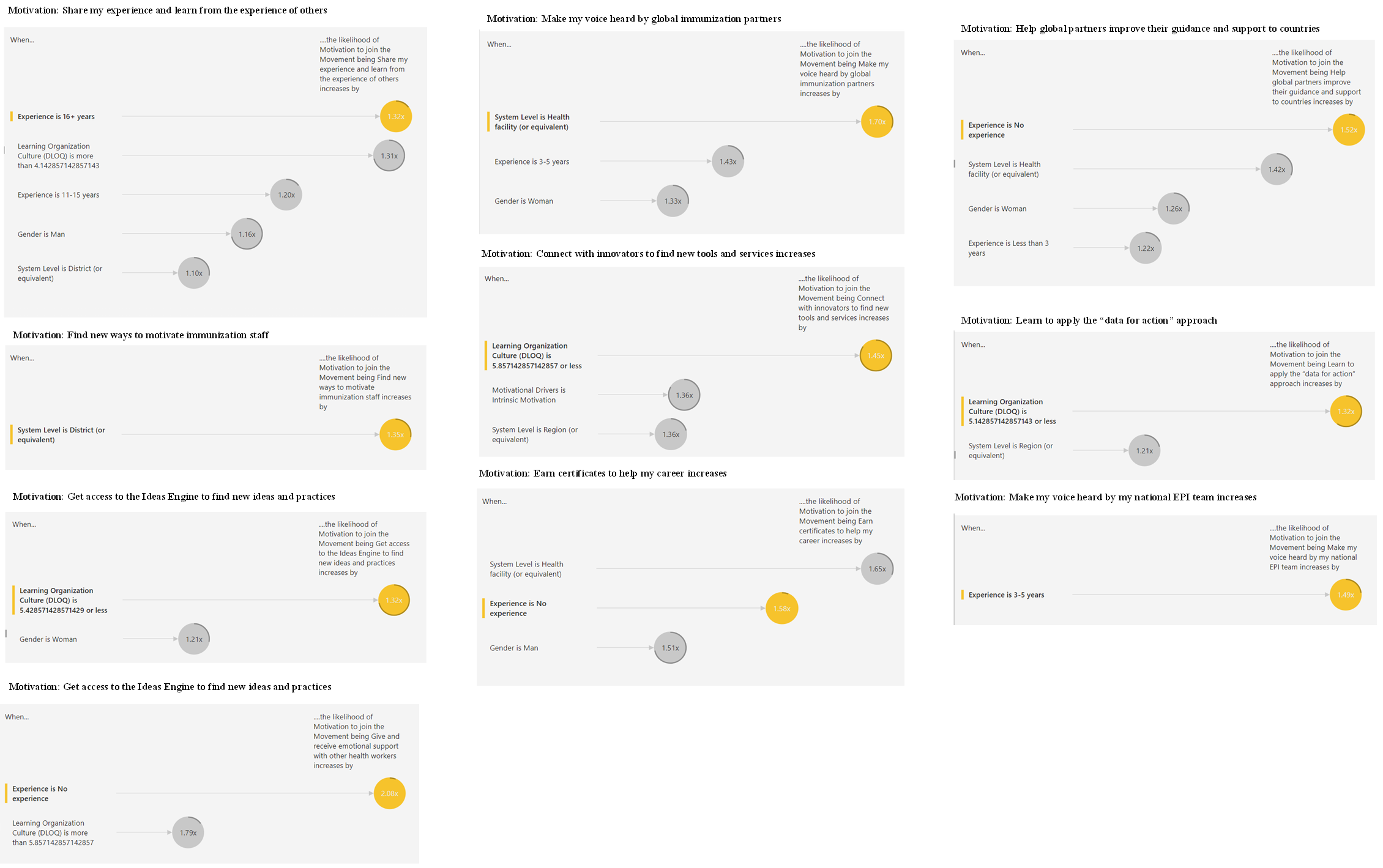

Supplement: S1 Fig — (TIF) [file pgph.0003855.s004.tif]
